# SIRT1 promotes proliferation and inhibits the senescence-like phenotype in human melanoma cells

**DOI:** 10.18632/oncotarget.1791

**Published:** 2014-02-19

**Authors:** Mickaël Ohanna, Caroline Bonet, Karine Bille, Maryline Allegra, Irwin Davidson, Philippe Bahadoran, Jean-Philippe Lacour, Robert Ballotti, Corine Bertolotto

**Affiliations:** ^1^ INSERM, U1065 (équipe 1), Equipe labélisée Ligue Contre le Cancer, C3M, Nice, France.; ^2^ Université de Nice Sophia-Antipolis, UFR Médecine, Nice, France.; ^3^ Institut de Génétique et de Biologie Moléculaire et Cellulaire, CNRS, INSERM, Université de Strasbourg, Illkirch, France.; ^4^ Centre Hospitalier Universitaire, Service de Dermatologie, Nice, France

**Keywords:** melanoma, MITF, SIRT1, PLX4032, treatment

## Abstract

SIRT1 operates as both a tumor suppressor and oncogenic factor depending on the cell context. Whether SIRT1 plays a role in melanoma biology remained poorly elucidated. Here, we demonstrate that SIRT1 is a critical regulator of melanoma cell proliferation. SIRT1 suppression by genetic or pharmacological approaches induces cell cycle arrest and a senescence-like phenotype. Gain and loss of function experiments show that M-MITF regulates SIRT1 expression, thereby revealing a melanocyte-specific control of SIRT1. SIRT1 over-expression relieves the senescence-like phenotype and the proliferation arrest caused by MITF suppression, demonstrating that SIRT1 is an effector of MITF-induced proliferation in melanoma cells. Interestingly, SIRT1 level and activity are enhanced in the PLX4032-resistant BRAF^V600E^-mutated melanoma cells compared with their sensitive counterpart. SIRT1 inhibition decreases melanoma cell growth and rescues the sensibility to PLX4032 of PLX4032-resistant BRAF^V600E^-mutated melanoma cells. In conclusion, we provide the first evidence that inhibition of SIRT1 warrants consideration as an anti-melanoma therapeutic option.

## INTRODUCTION

The class III histone deacetylases known as sirtuins has been associated with longer lifespan in yeast and worms. The mammalian ortholog SIRT1 has emerged as an important regulator of cancer and ageing. SIRT1 controls gene expression, cell cycle regulation, apoptosis, DNA repair, metabolism and senescence. The role of SIRT1 is however somewhat puzzling, acting both as a tumor suppressor or tumor promoter. It is thought that the precise role of SIRT1 may depend on the specific cell or tumor type and the presence or absence of p53 [1]. This tissue-specificity role might also involve, if any, tissue-specific regulation. A better understanding of the role of SIRT1 in specific tissues will provide the molecular basis for development of novel anti-aging and anti-cancer therapeutic targets. Up to now, the role of SIRT1 in melanoma is unknown.

Melanoma is a very aggressive neoplasm, well-known for its resistance to apoptotic stimuli. Apoptotic resistance represents an important cause, which limits the efficacy of the anti-melanoma therapies developed so far. Senescence is another important cellular failsafe mechanism, which is characterized by a state of stable cell cycle arrest. Senescence arises ordinarily in normal cells in response to telomere erosion or to oncogenic stresses. Although most cancer cells have conceivably bypassed OIS, several lines of evidence recently indicated that cellular senescence remains latently functional and can be reactivated in cancer cells, including melanoma cells.

Microphthalmia-associated transcription factor (M-MITF) is a melanocyte lineage-specific transcription factor of the c-myc supergene family. Its role in melanocyte physiopathology is complex. C. Goding and co-workers proposed that expression level, post-translational modification and co-factors, create a bar code-like situation which channels MITF towards a specific subset of target genes and determines MITF activity according to the cell context [2, 3]. Hence, MITF plays a critical role in melanocyte development [4] and functioning [5, 6] but it is also considered a bona fide melanoma oncogene. Indeed, genomic amplification of MITF associated with a decreased five-year survival [7] and germline mutation that predisposes carriers to melanoma [8-10] were reported. Understanding the molecular mechanisms underlying the role of MITF according to the melanocyte context is critically required.

Here, we observe an increased SIRT1 activity in human melanoma cells compared with normal human melanocytes. We demonstrate that SIRT1 suppression induces a senescence-like phenotype and its associated cell proliferation arrest. Based on the observation that SIRT1 suppression mimics some of the MITF knock-down effect, we investigated the epistatic relationship between MITF and SIRT1. Our results reveal that MITF controls SIRT1 expression at the transcriptional level. Moreover, SIRT1 overexpression relieves the senescence-like phenotype and the proliferation arrest caused by MITF knock-down, thereby demonstrating that SIRT1 is an effector of MITF-induced proliferation in melanoma cells. Our results reveal that SIRT1 activity is higher in PLX4032-resistant BRAF^V600E^-mutated melanoma cells compared with their sensitive counterpart. Furthermore, SIRT1 level and activity are dramatically inhibited in BRAF^V600E^-mutated melanoma cells sensitive to PLX4032, whereas they remain elevated in their resistant counterpart. Most importantly, SIRT1 inhibition rescues the sensitivity to PLX4032 of the resistant BRAF^V600E^-mutated melanoma cells.

## RESULTS

### SIRT1 activity is elevated in melanoma cells

**Figure 1:**
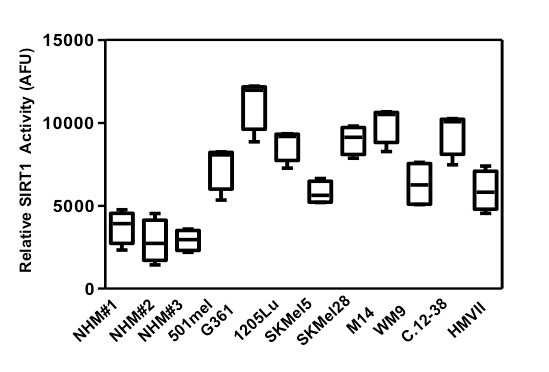
SIRT1 activity is elevated in melanoma cells SIRT1 activity was determined in melanoma cells of different genetic background and in normal melanocytes using an *in vitro* deacetylation assay. *Relative SIRT1 activity* is *expressed* as *arbitrary fluorescence units* (*AFU*). Values are expressed as box-and-whisker plots (n=3).

SIRT1 and AMPK are two energy metabolic sensors, and metabolism alteration is a crucial hallmark of *cancer* [11]. Whereas AMPK has been recently implicated in melanoma disease [12, 13], the role of SIRT1 has never been investigated. To determine the role of SIRT1 we first assessed SIRT1 activity in melanoma cell lines and in cells freshly isolated from human biopsies, and in normal human melanocytes. To this aim, we assessed the degree of deacetylation of a substrate which represents a peptide containing amino acids 379-382 of human p53 (Arg-His-Lys-Lys[Ac]), an established target of SIRT1 activity. The results showed an increased deacetylation of the p53 peptide in several melanoma cells of different genetic backgrounds compared with three different cultures of normal human melanocytes (Figure 1).

These results indicate that SIRT1 activity is enhanced during melanocyte transformation.

### SIRT1 suppression promotes a senescence-like phenotype and its associated cell proliferation arrest

**Figure 2:**
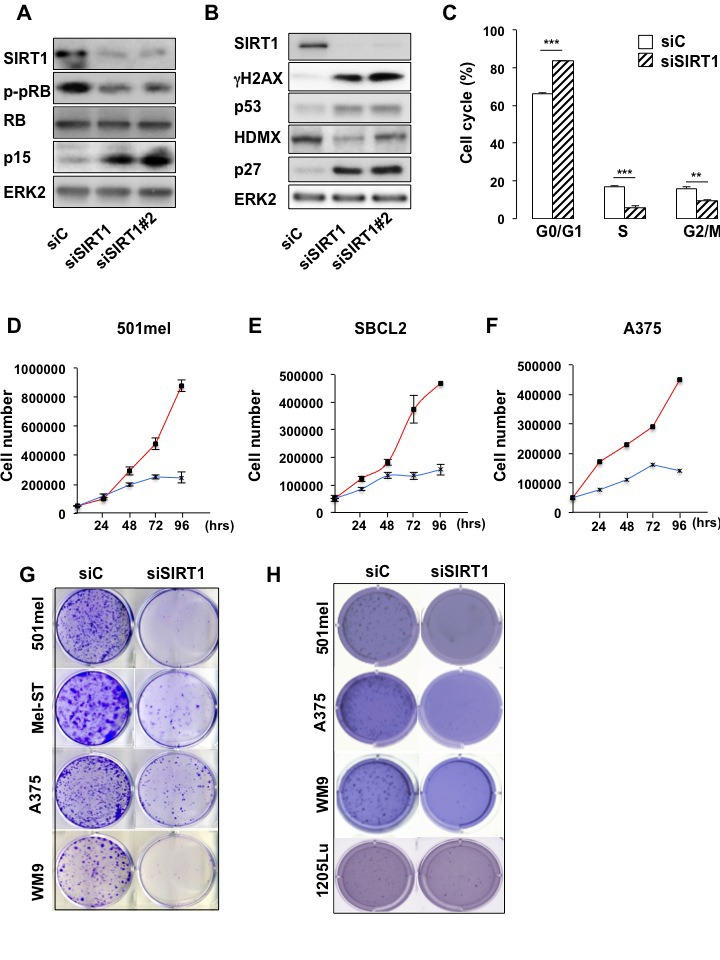
SIRT1 suppression triggers growth arrest (A-B) Western blot analysis of 501mel cells transfected with a control siRNA (siC) or two SIRT1 specific siRNAs (siSIRT1 and siSIRT1#2) for 96 hrs. (C) The cell cycle was analysed by FACS in 501mel cells transfected with control or SIRT1 siRNA for 96 hrs. Shown are the average values and standard deviations of three independent experiments. p-values of <0,01 (**) and of <0,001 (***) were considered statistically significant. (D-F) 501mel, SBCL2 and A375 human melanoma cells were plated in 6-well dishes, transfected with control (red line), or SIRT1 (blue line) siRNA and counted in triplicates from days 2 to 4. (G) Colony formation of 501mel cells transfected with control, or SIRT1 siRNA was assessed after 14 days. Representative images of colonies formed are shown. (H) Same as (G) but examined for anchorage-independent growth (polyHema). Colony formation was assessed after 21 days. Representative images of colonies formed are shown.

Based on these observations, we investigated the impact of SIRT1 suppression in 501mel melanoma cells with specific siRNA, which triggered an efficient SIRT1 inhibition (Figure 2A). SIRT1 knock-down was associated with an increase in the level of the cell cycle inhibitors p27^KIP1^, p15 and the tumor suppressor p53 and with a decreased expression of HDMX the p53 regulator (Figures 2A-B). Accordingly, SIRT1 suppressed melanoma cells displayed an hypophosphorylation of the retinoblastoma protein (Figures 2A-B), and a G0/G1 cell cycle arrest (Figure 2C). Accordingly, SIRT1-suppressed 501mel cells stopped proliferating after 72 hrs compared with cells transfected with a control siRNA (Figure 2D). The proliferation arrest also translated into a reduced ability to form anchorage-dependent and independent colonies (Figures 2G-H). Reduction in cell proliferation and/or colony formation was observed in several melanoma cell lines and in cells freshly isolated from human biopsies harboring the BRAF^V600E^ mutation (501mel, A375, WM9 and C.09-02) or the NRAS^Q61K/R^ mutation (HMVII and C.12-38) or in cells wild-type for BRAF and NRAS (SBCL2, Mel-ST) (Figures 2D-H, S1A-B). Collectively, these observations indicate that SIRT1 silencing promotes a cell cycle arrest in melanoma cells of different genetic background. To rule out the possibility of non-specific effect, we used a second siRNA (siSIRT1#2), both siRNA suppressed SIRT1, elevated the level of cell cycle inhibitors (Figure 2A-B) and reduced cell proliferation (Figure S1A) to similar extent.

We next determined the underlying mechanisms by which SIRT1 suppression caused cessation of cell proliferation in melanoma cells. SIRT1 silencing was reported to induce senescence or apoptosis depending on the cell type [14]. Melanoma cells from the above experiments showed no morphological sign of cell death, prompting us to argue that SIRT1 knock-down would trigger a senescence-like phenotype in melanoma cells.

**Figure 3:**
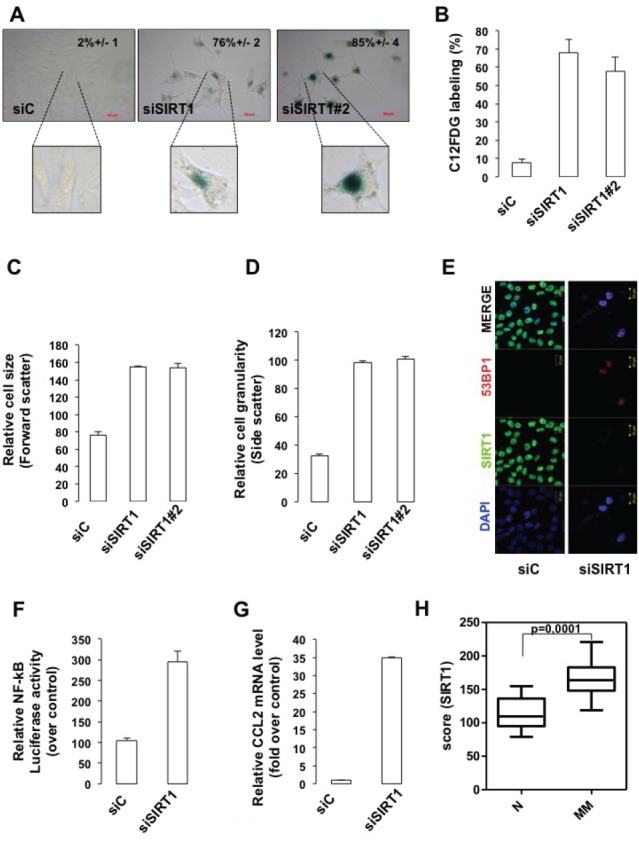
SIRT1 suppresses senescence (A) 501mel cells were transfected with control (siC) or two SIRT1 siRNA (siSIRT1 and siSIRT1#2) for 96 hrs and were stained for SA-βGal activity. The percentage of means and standard deviations (+SD) of β-Galactosidase positive cells were derived from counting 100 cells in duplicate plates. Enlargement of the cell is shown. (B) Same as (A) but examined for *C12FDG* staining. *Mean values* + *SD.* (C) The relative size (forward scatter) and (D) relative cell granularity (side scatter) of control or SIRT1-suppressed 501mel cells were analyzed by flow cytometry. Shown are the results of two independent experiments. (E) Immunofluorescence analysis with antibody to SIRT1 and 53BP1 of cells transfected with control (siC) or SIRT1 siRNA for 96 hrs. (F) NF-kB luciferase activity of 501mel cells transfected with control (siC) or SIRT1 siRNA for 96 hrs. (G) CCL2 mRNA level analysed by QRT-PCR in 501mel cells transfected with control or SIRT1 siRNA. (H) SIRT1 level in a subset of nevi and primary melanomas. The dataset was previously published under GSE46517.

Compared with control siRNA, the two different SIRT1 siRNA promoted a 70-80% increase in the senescence-associated β-galactosidase (SA-βGal) biomarker detected either by histochemical staining (Figure 3A) or FACS analysis (Figure 3B). In addition, an increase in cell size (Figure 3C) and in cell granularity (Figure 3D) were observed. Immunofluorescence studies showed that SIRT1-suppressed cells engaged the DNA damage response, a signaling pathway commonly associated with senescence, as illustrated by an induction in γH2AX and the detection of 53BP1 foci (Figures 2A and 3E). Likewise, SIRT1 suppression induced senescence-like phenotypes in different melanoma cells lines (Figure S1C). Senescence in melanoma cells is associated with production of a NF-κB-dependent secretome, which contains the chemokine CCL2 [15]. We used a vector containing NF-κB response elements that drives downstream transcription of the *luciferase* reporter gene. The increased luciferase activity in response to SIRT1 siRNA reflected an activation of the NF-κB signaling pathway (Figure 3F). Production of the senescence-associated secretory phenotype revealed by increased CCL2 mRNA expression was also observed (Figure 3G). Our findings *were* further substantiated using pharmacological inhibitors of SIRT1 (sirtinol, EX-527), which enhanced histone H3 acetylation on lysine 9 (H3K9Ac), another well-known SIRT1 substrate, and engendered similar level of SA-βGal stained cells (Figures S2B-D). We next sought to determine the relevance of SIRT1 in vivo. We *compared the SIRT1 expression profile* in a previously published dataset [16]. The analysis disclosed a significant lower expression of SIRT1 in nevi, benign melanocytic lesions compared with melanomas (Figure 3H). Collectively, reduction in SIRT1 level is associated with a decrease in cell proliferation and with traits of cellular senescence.

### MITF regulates SIRT1 deacetylase activity

**Figure 4:**
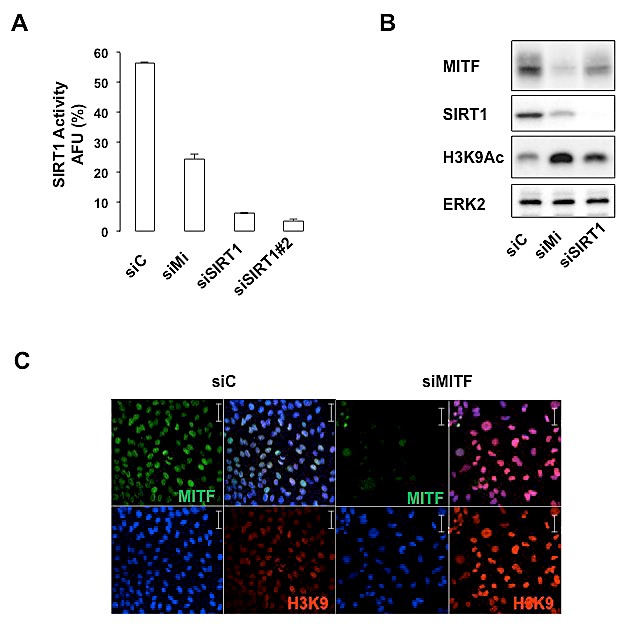
MITF regulates the activity of SIRT1 (A) *In vitro* deacetylation assay in cells transfected with control, MITF or two different SIRT1 siRNA. Activity is expressed as the *percentage* of activity with respect to the control. AFU, A*rbitrary fluorescence units*. Error bar represents the SEM of triplicate experiments. (B) Western blot analysis of 501mel cells transfected with control (siC), MITF (siMi) or SIRT1 siRNA. (C) Same as (B) but examined by immunofluorescence with anti-MITF and anti-histone H3K9 acetyl antibody.

Interestingly, we noticed that the senescence effects triggered by SIRT1 suppression in 501mel cells partly overlaped those of MITF knock-down. We therefore asked whether MITF could affect SIRT1 activity. We observed that MITF suppression by siRNA significantly reduced (about 50%) the level of deacetylated p53 peptide, indicating that MITF suppression reduced the activity of SIRT1. As a positive control, we measured SIRT1 activity in SIRT1-suppressed cells by two different SIRT1 siRNA that led to an almost complete inhibition of p53 peptide deacetylation (Figure 4A). MITF suppression also increased histone H3 acetylation on lysine 9 as judged by western blotting (Figure 4B) or immunofluorescence (Figure 4C) experiments. In aggregates, the results demonstrate that MITF regulates the activity of SIRT1, which is accompanied by a change in the acetylation status of its downstream target such as p53 and histone H3.

### MITF controls the level of SIRT1

**Figure 5:**
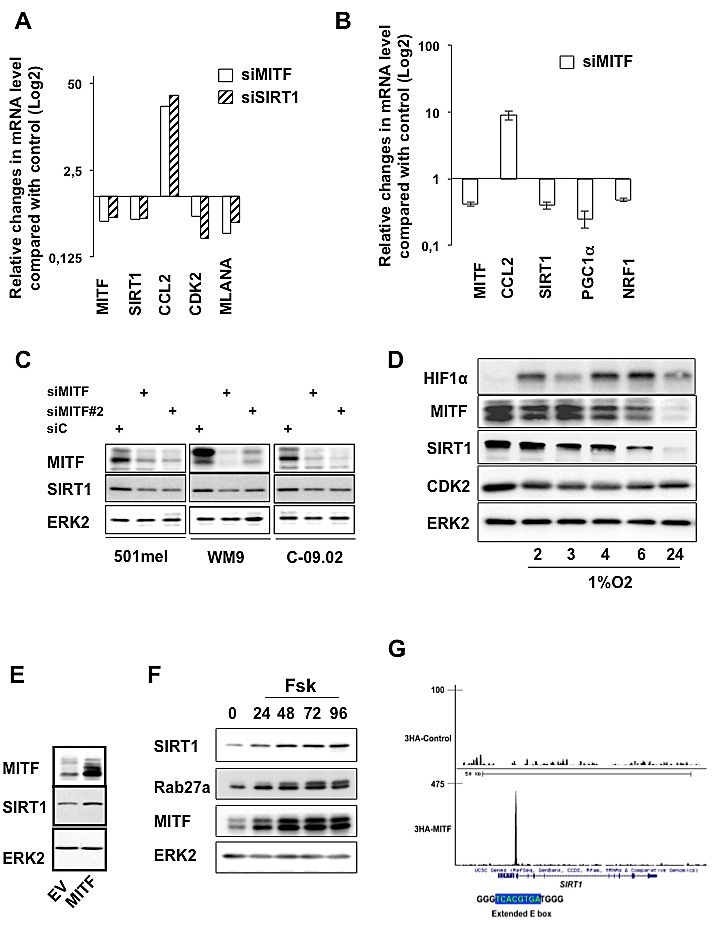
MITF regulates SIRT1 expression at the transcriptional level (A) qRT-PCR analysis of the genes indicated on the figure in 501mel melanoma cells transfected with control, MITF or SIRT1 siRNA for 96 hrs. Relative changes in mRNA level compared with control (Log2) is shown. (B) qRT-PCR analysis of the genes indicated on the figure in 501mel melanoma cells transfected with control or MITF siRNA for 96 hrs. Relative mRNA level is shown. (C) Western blot analysis of melanoma cells transfected with control or two different MITF siRNA. (D) 501mel cells were exposed to hypoxia (1% O2) for the time indicated and lysates were analysed by western blot. (E) Western blot analysis of 501mel cells transduced with control or MITF encoding adenovirus. (F) 501mel cells were exposed to forskolin (20 μM) for the time indicated and lysates were analysed by western blot. (G) UCSC view of 3HA-tagged wild-type MITF occupancy at the SIRT1 locus in 501mel cells. The sequence of the E box and promoter (blue) are shown.

Having shown that MITF suppression impaired SIRT1 activity, we investigated the impact of MITF on SIRT1 expression. In support of this idea, previous transcriptomic analysis suggested that, among members of the sirtuin gene family, only SIRT1 was decreased in MITF silenced cells (Figure S3A). QRT-PCR experiments in two melanoma cell lines (501mel, WM9) and in cells freshly isolated from a human biopsy (C-09.02) showed, as expected, that MITF suppression by siRNA, compared with control cells, caused a decreased mRNA expression of two of its target genes, MLANA and CDK2 [17] and an increased mRNA expression of CCL2, as previously reported (Figures 5A and S3B-C) [15]. mRNA expression of SIRT1 was decreased in MITF-silenced cells compared with control cells and was associated with a concomitant change in genes involved in mitochondrial biogenesis, such as PGC1α and one of its target gene NRF1 (Figure 5B).

Western blot analysis confirmed that MITF knock-down with two different siRNA correlated with a substantial reduction in SIRT1 level (Figure 5C). Likewise, immunofluorescences showed that SIRT1 mainly localized to the nucleus in melanoma cells and that SIRT1 reduction paralleled that of MITF (Figure S3D). We next assessed whether reduction of MITF by hypoxia, a situation commonly found in tumors, also triggered a change in SIRT1 level. Kinetics of hypoxia, as judged by the stabilization of HIF1α, reduced MITF expression [18] and one of its target gene CDK2 [17] as previously reported respectively, which was also accompanied by a decreased expression of SIRT1 (Figure 5D). Conversely, forced expression of MITF caused elevation of SIRT1 expression (Figure 5E). Moreover, in condition of MITF regulation via activation of the cAMP-pathway [5], the known MITF target RAB27a [19] and SIRT1 expression were enhanced as a function of time (Figure 5F). These results strongly suggested the existence of an epistatic relationship between MITF and SIRT1. We therefore searched in our previously published MITF-ChIP-seq data for genomic sites bound by MITF in the SIRT1 locus [20]. Interestingly, the site bound by MITF comprises an extended palindromic E box sequence (5'-TCACGTGA) characteristic of the MITF binding sites (Figure 5G and S4A). In silico analysis revealed that this promoter sequence is highly conserved during evolution (Figure S4B). In conclusion, our results indicate that MITF controls SIRT1 expression at the transcriptional level and point out to a melanocyte-specific regulation of SIRT1.

### SIRT1 mediates MITF effect

**Figure 6:**
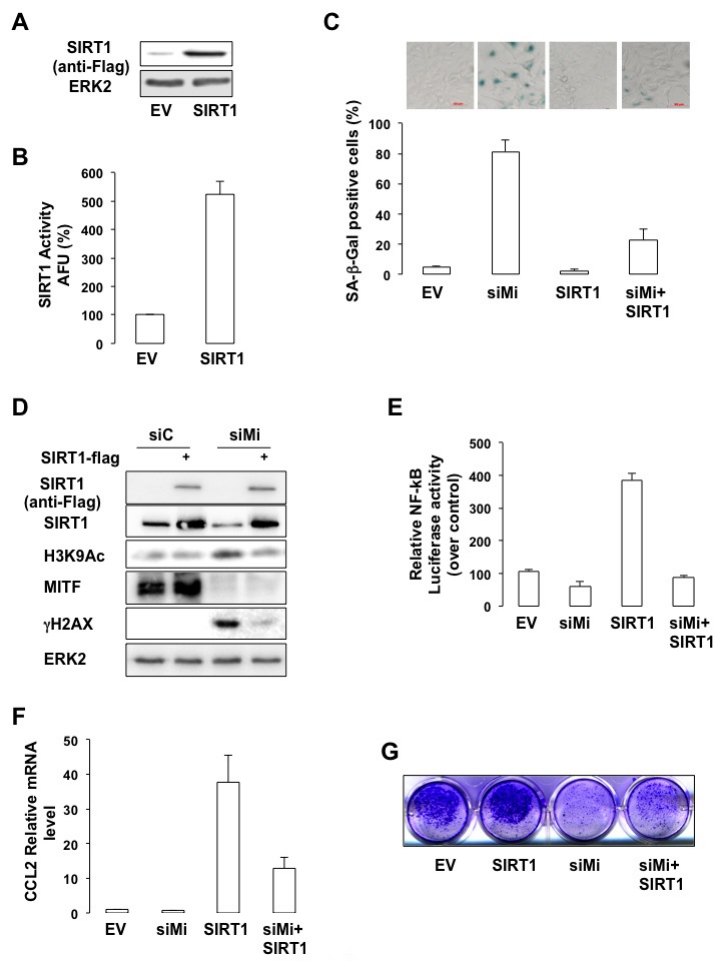
SIRT1 prevents the senescence phenotypes caused by MITF suppression (A) Western blot analysis of cells transfected with a vector encoding FLAG-tagged SIRT1. (B) *In vitro* deacetylation assay in cells transfected with an empty vector (EV) or a vector encoding FLAG-tagged SIRT1. Activity is expressed as the *percentage* of activity with respect to the control (EV). AFU, A*rbitrary fluorescence units*. Error bar represents the SEM of triplicate experiments. (C) 501mel cells were transfected with control (siC) or MITF siRNA and/or an empty vector or a vector encoding FLAG-tagged SIRT1 for 96 hrs. Cells were stained for SA-βGal activity (x10 magnification). Results are expressed as the *mean* ± the *standard deviation* of three experiments of SA-βGal positive cells. (D) Same as © but analysed by western blot. (E) Control or MITF-suppressed 501mel cells were transfected with κB-Luc *reporter plasmid* plus an empty vector or a vector encoding FLAG-tagged SIRT1 for 96 hrs. NF-kB luciferase activity is expressed relative to control cells. (F) Same as (C) but examined for expression of the CCL2 mRNA. (G) Colony formation of 501mel cells transfected with control, or MITF siRNA ± a vector encoding FLAG-tagged SIRT1 was assessed after 14 days. Representative images of colonies formed are shown.

We next sought to determine the importance of SIRT1 in the senescence phenotype mediated by MITF suppression. SIRT1 forced expression (Figure 6A), led to an increased SIRT1 activity (Figure 6B), and prevented SA-βGal staining mediated by MITF-suppression in melanoma cells (Figure 6C). In addition, the increase in H3K9 acetylation and in the level of γH2AX observed in response to MITF-suppression was dramatically reduced upon SIRT1 forced expression (Figure 6D). Likewise, SIRT1 forced expression strongly reduced NF-κB activation (Figure 6E) and production of CCL2 (Figure 6F) mediated by MITF knock-down. Finally, SIRT1 forced expression partially rescued the ability of MITF-deleted 501mel cells to form colonies (Figure 6G). In conclusion, our results indicate that SIRT1 acts downstream of MITF in the regulation of melanoma cell proliferation.

### SIRT1 mediates PLX4032 resistance

**Figure 7:**
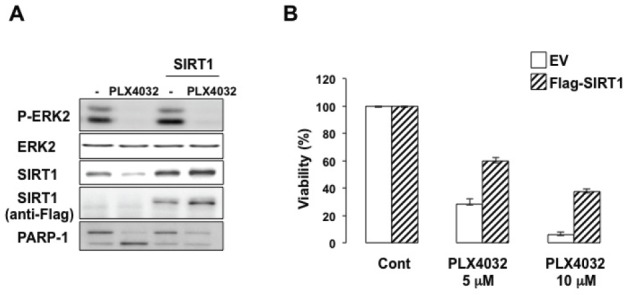
SIRT1 contributes to PLX4032 resistance (A) 501mel melanoma cells control or transfected with a FLAG-tagged SIRT1 were exposed to PLX4032 (5μM) for 96 hrs. Lysates were analyzed by western blotting. (B) 501mel melanoma cells control or transfected with a FLAG-tagged SIRT1 were exposed to PLX4032 (5μM or 10μM) for 48 hrs. Relative cell viability was expressed as a percentage (%) of the control cells (EV).

**Figure 8:**
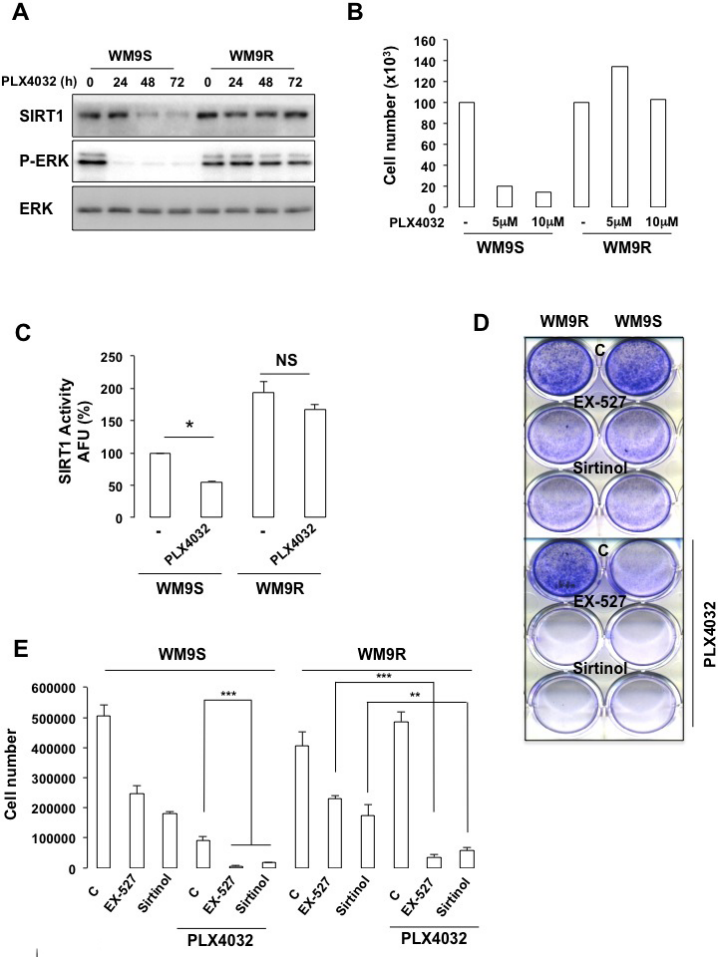
SIRT1 inhibition impairs melanoma cell growth and resistance to PLX4032 (A) *Western Blot analysis* of PLX4032 sensitive (WM9S) or resistant (WM9R) WM9 melanoma cells to PLX4032 5μM. (B) Number of WM9S and WM9R cells exposed to PLX4032 5μM and 10μM for 96 hrs. (C) *In vitro* deacetylation assay in WM9S and WM9R cells exposed to PLX4032 5μM for 96 hrs. (D) WM9S and WM9R melanoma cells were left in basal condition or exposed to SIRT1 inhibitors, EX-527 (34 μM) and sirtinol (163 μM) in presence or absence of PLX4032 5μM. Colonies were *stained with crystal violet* after 14 days. Representative images of colonies formed are shown. (E) Same as (D). Colonies were destained and the relative absorbance, which reflects the number of colonies was measured on a spectrophotometer.

MITF has been involved in PLX4032 resistance. We therefore asked whether SIRT1, a downstream MITF target, might contribute to this resistance. In BRAF^V600E^-expressing 501mel cells, PLX4032 inhibited ERK2 activation as shown by the decreased ERK2 phosphorylation, and induced cell death illustrated by the cleavage of PARP1 (Figure 7A) and the decrease in cell viability (Figure 7B). After 96 hrs, the decrease in cell viability (Figure 7B) was associated with a reduction in SIRT1 level (Figure 7A). However, it should be noted that short time treatment with PLX4032 (24 hrs), efficiently inhibited ERK2 phosphorylation but had no effect on SIRT1 level (Figure 8A) and was not associated with cell growth arrest or death (not shown). Moreover, forced expression of SIRT1 prevented the cleavage of PARP1 and rendered 501mel cells resistant to PLX4032 effect. Therefore, SIRT1 attenuates PLX4032 effect.

We next thought to determine the activity of SIRT1 in BRAF^V600E^-mutated WM9 melanoma cells sensitive (WM9S) to PLX4032 and in their resistant counterpart (WM9R). As expected, WM9S exposed to PLX4032 displayed almost no ERK phosphorylation and *no* change in levels of total *ERK*, which reflected an inhibition of ERK activity (Figure 8A). In WM9R, ERK2 phosphorylation remained elevated all along the time course. Consequently, PLX4032 dramatically reduced the number of cells (Figure 8B) and colonies (Figures 8D-E) in the WM9S but not in the WM9R. Interestingly, WM9R exhibited an enhanced SIRT1 level and activity compared with WM9S (Figures 8A and 8C). Moreover, SIRT1 expression and activity was strongly reduced after exposure to PLX4032 in WM9S, but not in WM9R, where SIRT1 expression and activity remained high (Figures 8A and 8C). We conclude that high SIRT1 correlates with PLX4032 resistance.

Using pharmacological approaches, we investigated whether inhibition of SIRT1 may improve the response to PLX4032. The SIRT1-specific inhibitor EX-527 [21] or sirtinol, a synthetic small-molecule inhibitor of SIRT1 and SIRT2 [22] suppressed proliferation as illustrated by a decreased number of cells and colony formed in WM9S and in WM9R (Figures 8D-E). Whereas PLX4032 had no effect in the resistant WM9R cells, a strong reduction in colony formation ability was achieved by PLX4032 in WM9S. However, PLX4032, and the SIRT1 inhibitors, had a synergistic effect leading to an almost complete inhibition of cell proliferation in both WM9S and WM9R. Moreover, these data indicated that SIRT1 inhibition rescued the sensitivity to PLX4032 of WM9R.

In conclusion, these results support the idea that SIRT1 plays a role in the resistance to PLX4032 of melanoma cells and that a combination therapy consisting of PLX4032 and SIRT1 inhibitors represents a valuable therapeutic option.

## DISCUSSION

This study was designed to investigate the role of SIRT1 in melanoma cells. Here, we demonstrate that SIRT1 suppression by genetic or pharmacological approaches triggers a cessation of cell proliferation and senescence-like phenotype. Indeed, in addition to the β-galactosidase activity at pH6, which although imperfect is the most widely used marker of senescence, SIRT1-suppressed melanoma cells display changes in morphology characterized by an increase in cell size and granularity, and an increase in the level of cell cycle inhibitors.

These effects that are observed in several melanoma cell types or cells freshly isolated from human biopsy of different genetic background, link SIRT1 to melanoma cell proliferation. We also find that senescence in SIRT1-suppressed cells is accompanied by a decrease expression in HDMX. In line with this, HDMX knock-down promoted senescence in human prostate adenocarcinoma cells [23].

Our findings reveal for the first time a lineage-specific control of SIRT1 expression. The M-isoform of MITF is specifically expressed in melanocytes [24] and nearly all melanoma cells express MITF [25]. Our observations indicate that MITF regulates expression of SIRT1 at the transcriptional level, as evidenced by a change in SIRT1 mRNA and protein level and ChIP-seq data showing binding of MITF to the promoter of SIRT1. Analysis of the SIRT1 promoter reveals the presence of a MITF-binding site composed of 5'-TCACGTGA-3'. This sequence matches perfectly the MITF consensus site previously reported, with a T in 5' and an A in 3' [26]. Moreover, we found a paralleled expression of MITF and SIRT1 in response to stimuli involved in melanocyte differentiation (cAMP-elevating agents) and in melanomagenesis (hypoxia). It would be interesting to determine if SIRT1 indeed mediates the effect of MITF in these contexts.

Some melanoma cell lines displays very low MITF level. As MITF has been shown both in vitro and in vivo to be critical to melanoma cell proliferation/survival [4, 27], A375 or 1205Lu cells with very low level of MITF have likely adapted to the lost of MITF.

One explanation for the lack of correlation between MITF and SIRT1 in such cells is that, SIRT1 expression is regulated by other transcription factors, which activities might be influenced by the genetic background of the cells, previously reported to control SIRT1 expression [28]. Another explanation is that SIRT1 expression might be regulated by transcription factors homologous to MITF, such as TFE3 or TFEB. Both hypotheses remain to be investigated.

Whereas, SIRT1 null mice were embryonic lethal [29], Sirt1^+/-^ heterozygous mice show signs of premature aging, such as graying coat. In this mouse model, hair graying has been shown to result from defective self-maintenance of melanocyte stem-cells within the niche [30]. Hair graying is also observed in the Mi^vit/vit^ mouse model [31], which expresses the D222N substitution in the transcription factor MITF and causes a progressive loss of the melanocytes [24]. Whether SIRT1 expression is decreased in the melanocyte stem cells in vivo, and contributes to the melanocyte lost, remains to be determined. Nevertheless, the data from both our in vitro results and from in vivo models support the idea that SIRT1 activity is critically required for melanocyte cell proliferation.

The regulation of SIRT1 by MITF suggests the existence of other tissue-restricted mechanisms of SIRT1 regulation. These tissue-specific regulations would contribute to SIRT1 activity in a tissue-specific manner and might explain the apparent discrepancies regarding SIRT1 function in different cell types.

SIRT1 has been shown to control the *MAPK/ERK signaling pathway* [32] and vice versa [33-35]. In our model system, neither forced expression of SIRT1 (Figure 7A) nor short-term SIRT1-suppression affect the level of phospho-ERK (data not shown). These observations indicate that SIRT1 does not regulate the MAPK/ERK signaling pathway at least in the melanoma cell line used in this study. Noteworthy, SIRT1 level decreases in PLX4032-treated melanoma cells after 96 hours (Figure 7A). However, a shorter treatment with PLX4032 (Figure 8A, lane 2) efficiently inhibits ERK2 phosphorylation, but it has no effect on SIRT1 level. In this later condition, we also observe no growth arrest or cell death (not shown).

However, longer exposure to PLX4032 (Figure 7A and 8B left part), reduces the number of cells and is associated with a decrease in SIRT1 level. In aggregates, our results suggest that SIRT1 level may be reduced as a result of growth arrest/cell death mediated by the inhibitors of the BRAF/MEK/ERK pathway. This is in agreement with previous reports showing a reduction in SIRT1 level ensuing caspase activation [33, 35].

Our results show that SIRT1 over-expression rescued melanoma proliferation arrest mediated by MITF knock-down, thereby indicating that SIRT1 is an effector of MITF-induced cellular proliferation. Therefore, deregulation of SIRT1 might play an important role in melanoma pathogenesis. Recently, MITF was reported to contribute to PLX4032 resistance [36]. Moreover, MITF through regulation of PGC1α, has been shown to mediate a metabolic rewiring that limits the efficacy of BRAF inhibitors [37, 38]. Importantly, SIRT1 regulates PGC1αacetylation which is thought to be crucial for its activity [39, 40]. These data suggest that MITF might *coordinate* the expression of different proteins involved in oxidative metabolism in melanoma cells. Moreover, PGC1α has been shown to limit the efficacy of PLX4032 [37] and to mediate the resistance to chemotherapy drug [41]. We find an increased activity of SIRT1 in PLX4032 resistant BRAF^V600E^-mutated melanoma cells compared with their sensitive counterpart, thereby linking SIRT1 to drug resistance and poor prognosis in melanoma. In that context, we demonstrate that inhibition of SIRT1 with genetic or pharmacological approaches additively enhances the efficacy of PLX4032 of melanoma cells but more importantly that it rescues the sensitivity to PLX4032 of the resistant BRAF^V600E^-mutated melanoma cells.

The findings gathered in this study not only contribute to better understand the role of MITF in melanoma cells but also provide strong support that targeting SIRT1 is a valuable clinical option to treat malignant melanoma.

## MATERIALS AND METHODS

### Cell cultures, transfection and luciferase activity

The human melanoma cells were grown in DMEM supplemented with 7% FBS at 37°C in a humidified atmosphere containing 5%CO2. 501mel, 1205Lu, A375, WM9 and SBCL2 are wild type for p53 and SKmel28 (L145R) and M14 (G266E) are p53 mutated. SBCL2 and WM9 cells were obtained from M. Herlyn. Fresh sterile tissues were obtained from surgical waste from patients diagnosed for metastatic melanoma at the Nice CHU hospital and treated as reported [27]. Informed consent was obtained from the patients.

For siRNA transfection, a single pulse of 50nM of siRNA was administrated to the cells at 50% confluency by transfection with 5μl lipofectamine™ RNAiMAX in opti-MEM medium (Invitrogen, San Diego, CA, USA). Control (siC) and MITF (siMi) siRNAs were previously described [42]. SIRT1 siRNA was from Sigma. For luciferase assay, cells were transiently transfected as previously described [5] using the lipofectamine reagent (Invitrogen).

### Senescence-Associated β-Galactosidase Assay

The senescence β-galactosidase staining kit from Cell Signaling Technology (Beverly, MA, USA) was used to histochemically detect β-galactosidase activity at pH6. The percentage of means and standard deviations were derived from counting 100 cells in duplicate plates after 96 hrs. Cells were also labeled with 20 nM C_12_FDG (Sigma) for 1 h. The cells were detached from the plate and analyzed by flow cytometry (MACSQuant® Miltenyi Biotech) using the fluorescein channel. Arbitrary units (median channel fluorescence) are reported.

### Western blot assays

Briefly, cell lysates (30μg) were separated by SDS-PAGE, transferred on to a PVDF membrane and then exposed to the appropriate antibodies, anti-MITF and anti- H3K9acetyl were from Abcam, anti-ERK2, anti-CDK2, and anti-p27^KIP1^ antibodies were from Santa Cruz Biotechnology, anti-SIRT1 and anti-phospho-ERK1/2 (Thr202/Tyr204) were from Cell Signaling Technology Inc. (Beverly, MA). Horseradish peroxidase-conjugated anti-rabbit or anti-mouse antibodies were from Dakopatts (Glostrup, Denmark). Proteins were visualized with the ECL system (Amersham). The western blots shown are representative of at least 3 independent experiments.

### Proliferation curves

Cells were seeded in 12-well dishes (10x10^3^ cells) and 48 hrs post-transfection, cells were trypsinized from days 2 to 6, counted in triplicate by haemocytometer to assess cell proliferation. The experiment was performed at least three times.

### Flow cytometry

Cells were stained with propidium iodide (40μg/ml) containing ribonuclease A (10μg/ml) and were analyzed using a fluorescence activated cell sorter (MACSQuant® Analyzer) and MACSQuantify™ software.

### Immunofluorescence and confocal microscopy

Immunofluorescence experiments were carried out as previously described [15] and examined with the 20X objective using Zeiss Axiophot microscope equipped with epifluorescence illumination.

### mRNA preparation, Real-time/quantitative PCR

mRNA isolation was performed with Trizol (Invitrogen), according to standard procedure. QRT-PCR was carried out with SYBR® Green I (Eurogentec, Seraing, Belgium) and Multiscribe Reverse Transcriptase (Applied Biosystems) and monitored by an ABI Prism 7900 Sequence Detection System (Applied Biosystems, Foster City, CA). Detection of RPL14 gene was used to normalize the results. Primer sequences for each cDNA were designed using either Primer Express Software (Applied Biosystems) or qPrimer depot (http://primerdepot.nci.nih.gov) and are available upon request.

### Colony-forming assays

Cells (2×10^3^) were seeded in 12-well dishes or on a layer of the nonadhesive 1% *polyHEMA* surface. The cells were then placed in a 37°C, 5% CO_2_ incubator. Colonies of cells were allowed to grow for 14 days. The colonies were stained with 0.04% crystal violet/2% ethanol in PBS for 30 min. Photographs of the stained colonies were taken. The colony formation assay was performed in duplicate.

### SIRT1 Activity

SIRT1 activity was measured by using the SIRT1 Assay Kit (Sigma). In this assay, SIRT1 activity is assessed by the degree of deacetylation of a substrate which represents a peptide containing amino acids 379-382 of human p53 (Arg-His-Lys-Lys[Ac]). SIRT1 activity is directly proportional to the degree of deacetylation of Lys-382. Nuclear cell lysates (quantity) were incubated with peptide substrate (25 μM) in a phosphate-buffered saline solution at 37°C for 45 minutes. The reaction was stopped with the addition of 2 mM nicotinamide and a developing solution that binds to the deacetylated lysine to form a fluorophore. Following 10 minutes incubation at 37°C, fluorescence was read in a plate-reading fluorometer at an excitation wavelength of 360 nm and an emission wavelength of 450 nm. In each assay, human recombinant SIRT1 enzyme (1 Unit per well), a SIRT1 activator, and suramin sodium (5 mM), a SIRT1 inhibitor were utilized as positive and negative controls in each set of reactions. A standard curve was constructed using deactylated substrate (0-10 μM). Data for endogenous SIRT1 activation were normalized to cellular protein concentration measured via BCA-assay.

## SUPPLEMENTARY FIGURES


