# Supplementary material for: SIRT1 promotes proliferation and inhibits the senescence-like phenotype in human melanoma cells
Source: Oncotarget. 2014 Feb 19;5(8):2085–95. doi: 10.18632/oncotarget.1791 (PMC4039147; doi:10.18632/oncotarget.1791)
Supplement: Supplementary file 1 [file oncotarget-05-2085-s001.pdf]

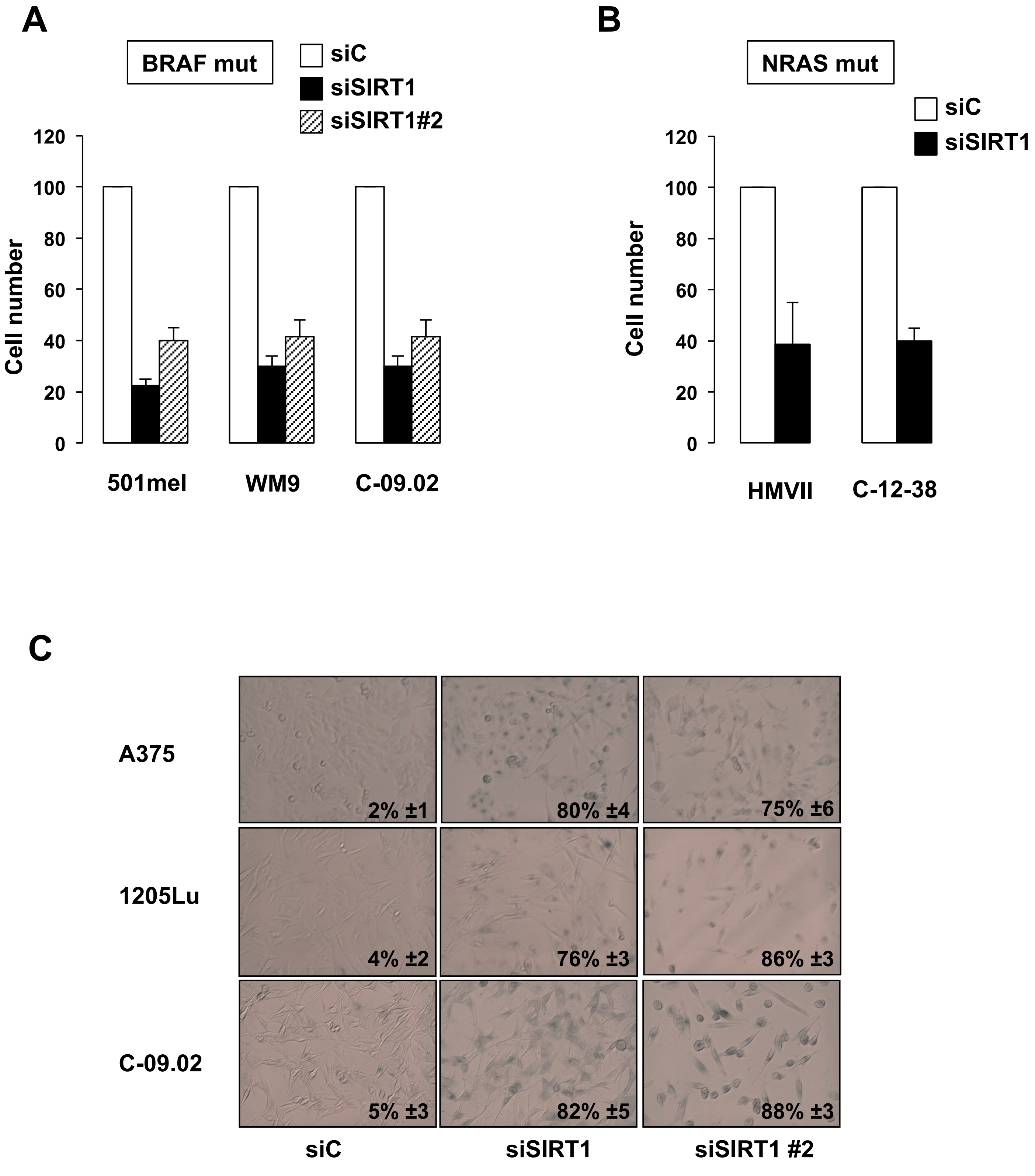

Supplementary Figure 1

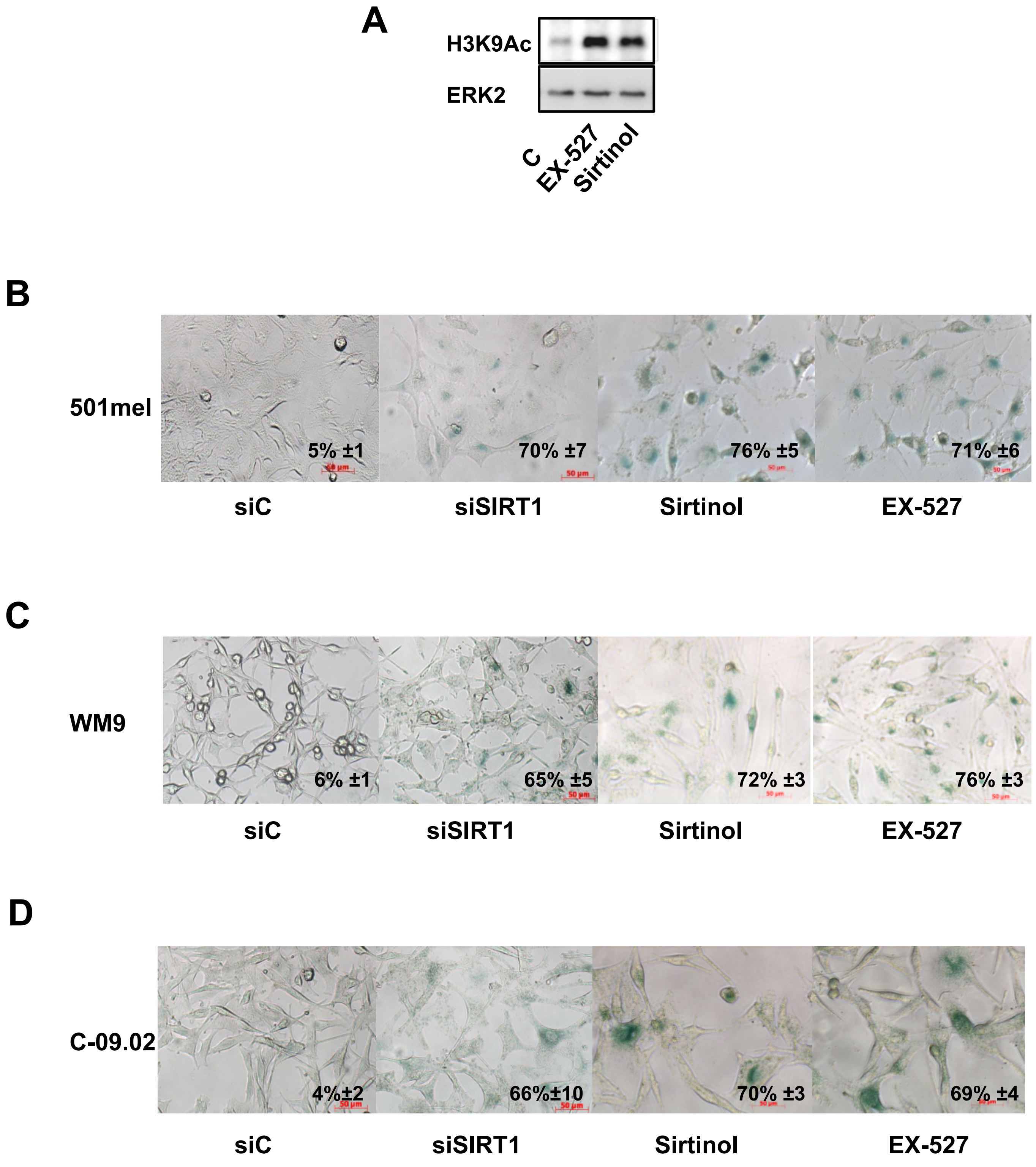

Supplementary Figure 2

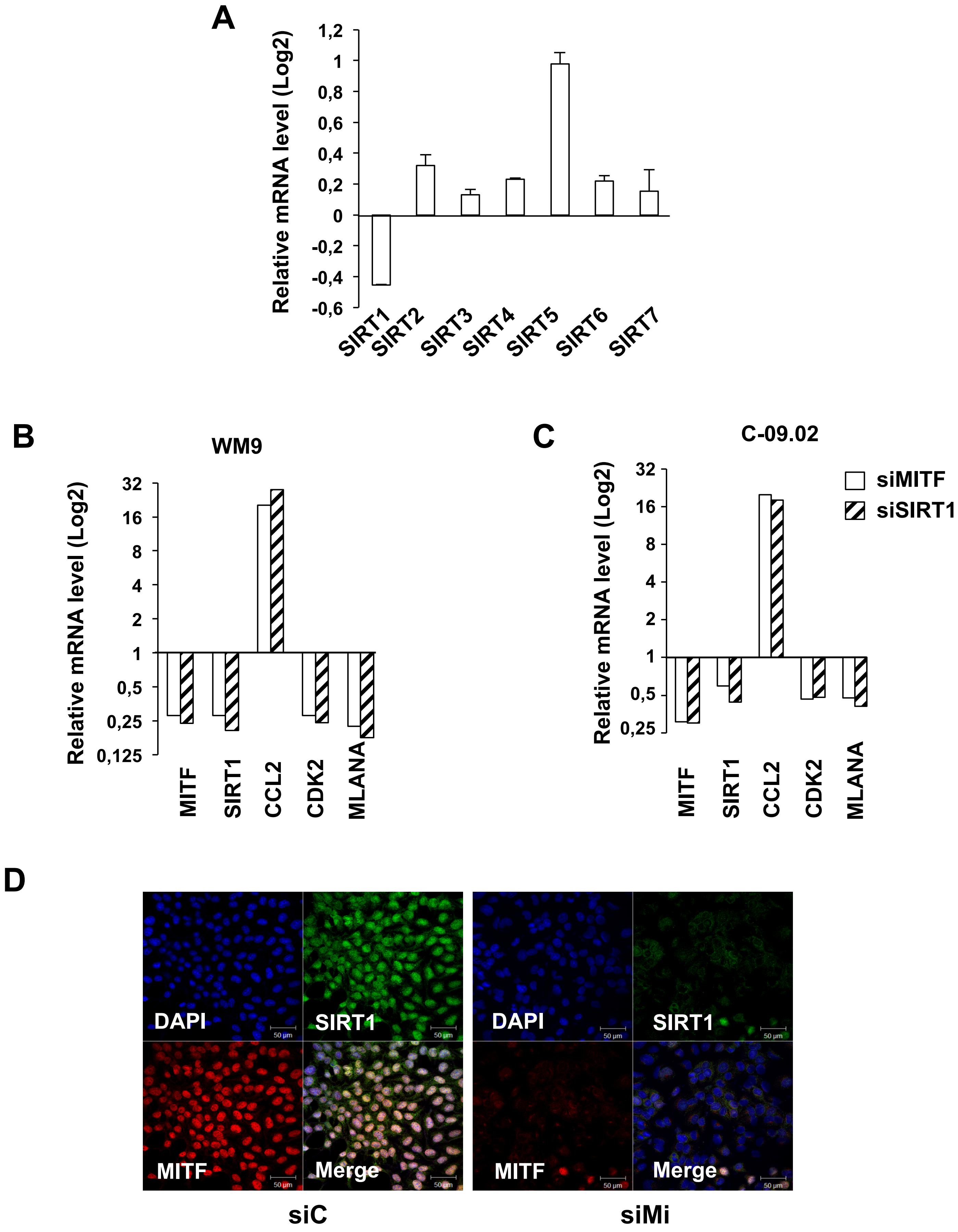

Supplementary Figure 3

A

Peak statistics from MACS output

Peak coordinates				
Chromosome	Beginning	End	Reads	Fold enrichment
10	69643848	69643848	283	436

B

```
Ornithorhynchus      -----------GGGGGCGGGGGGGGCGGTCACGTGA--CCCCTTTAAATCTCCCGCAGC--
Monodelphis          ------GGGAGGGGCCGACGGCGCGGGTCACGTGATGGGGGTTTAAATCTCCCGCAGCCG
Oryctolagus          GAGGGCGGGGGCGGGGGCGGAGGCAGGTCACGTGA-CGGGGTTTAAATCTCCCGCAGCCG
Bos                  --GAGGGCGGGGGCGAGGAGCGGCGCGTCACGTGA-CGGGGTTTAAATCTCCCGCAGCCA
Felis                --GAGGGCGGGGGCGAAGAGGGCCGGGTCACGTGA-CGGGGTTTAAATCTCCCGCAGCCC
Mus                  -GAGGCGCGGGGGCGGGGAGGGGCGGGTCACGTGA-CGGGGTTTAAATCTCCCGCAGCCG
Rattus               ------GCGGGGGCGGCGAGGGGCGGGTCACGTGA-CGGGGTTTAAATCTCCCGCAGCCG
Mustela              -GAGGGCGGGGGCGAAGA-GGACCGGGTCACGTGA-CGGGGTTTAAATCTCCCGCAGCCG
Homo                 -GAGGGCGGGGGCGGCGATGGGGCGGGTCACGTGA-TGGGGTTTAAATCTCCCGCAGCCG
Pan                  -GAGGGCGGGGGCGGCGATGGGGCGCGTCACGTGA-CGGGGTTTAAATCTCCCGCAGTCG
Cavia                ---GAGGGCGGGGGTGGCGAGGGGCGGTCACGTGA-CGGGGTTTAAATCTCCCGCAGCCC
Canis                ---GAGGGCGGGGGCGACGGGGCCGGGTCACGTGA-CGGGGTTTAAATCTCCCGCAGCCG
                                *       .      *********
```

Supplementary Figure 4

Supplementary figure legends

Figure S1. SIRT1 suppression triggers growth arrest and senescence-like phenotypes. (A) $BRAF^{V600E}$-mutated human melanoma cells (501mel, A375, C-09.02) were transfected with control or SIRT1 siRNA for 96 hrs and counted. **(B)** $NRAS^{Q61}$ mutated human melanoma cells (HMVII, C-12.38) were transfected with control or SIRT1 siRNA and were counted 96 hrs later. **(C)** SA-β-Gal staining of human melanoma cells transfected with control (siC) or SIRT1 siRNA for 96 hrs. Percentage of means and standard deviations of the percentages of SA-b-Gal positive cells are indicated (right).

Figure S2. Pharmacological inhibition of SIRT1 induces senescence traits. Human melanoma cells were transfected with control or SIRT1 siRNA or were exposed to sirtinol (163 μM) or EX-527 (34 μM), two pharmacological inhibitors of SIRT1 for 96 hrs. Percentage of means and standard deviations of SA-βGal positive cells are indicated.

Figure S3. MITF regulates SIRT1 expression at the transcriptional level. (A) Data sets of SIRT family members extracted from DNA microarray experiments of control or MITF-silenced 501mel melanoma cells [15]**. (B-C)** qRT-PCR analysis of melanoma cell lines (WM9) and cells freshly isolated from human biopsy (C-09.02). Relative mRNA level of cells transfected with MITF siRNA over cells tranfected with a control siRNA is shown. **(D)** Immunofluorescence analysis of control or MITF-suppressed 501mel cells with anti-MITF or anti-SIRT1 antibodies.

Figure S4. MITF binding to the promoter of SIRT1. (A) The peak coordinates and the fold enrichment value for MITF binding to the SIRT1 promoter, are shown. **(B)** Nucleotide sequence alignment of the proximal SIRT1 promoter region from several species shows that the E box motif is highly conserved.
